# Modeling Net Ecosystem Carbon Exchange of Alpine Grasslands with a Satellite-Driven Model

**DOI:** 10.1371/journal.pone.0122486

**Published:** 2015-04-07

**Authors:** Wei Yan, Zhongmin Hu, Yuping Zhao, Xianzhou Zhang, Yuzhi Fan, Peili Shi, Yongtao He, Guirui Yu, Yingnian Li

**Affiliations:** 1 Lhasa Plateau Ecosystem Research Station, Key Laboratory of Ecosystem Network Observation and Modeling, Institute of Geographic Sciences and Natural Resources Research, Chinese Academy of Sciences, Beijing 100101, China; 2 Graduate University of Chinese Academy of Sciences, Beijing 100049, China; 3 Beijing Meteorological Bureau, Beijing 100089, China; 4 Key Laboratory of Ecosystem Network Observation and Modeling, Institute of Geographic Sciences and Natural Resources Research, Chinese Academy of Sciences, Beijing 100101, China; 5 Northwest Institute of Plateau Biology, Chinese Academy of Sciences, Xining 810001, China; Peking University, CHINA

## Abstract

Estimate of net ecosystem carbon exchange (NEE) between the atmosphere and terrestrial ecosystems, the balance of gross primary productivity (GPP) and ecosystem respiration (Reco) has significant importance for studying the regional and global carbon cycles. Using models driven by satellite data and climatic data is a promising approach to estimate NEE at regional scales. For this purpose, we proposed a semi-empirical model to estimate NEE in this study. In our model, the component GPP was estimated with a light response curve of a rectangular hyperbola. The component Reco was estimated with an exponential function of soil temperature. To test the feasibility of applying our model at regional scales, the temporal variations in the model parameters derived from NEE observations in an alpine grassland ecosystem on Tibetan Plateau were investigated. The results indicated that all the inverted parameters exhibit apparent seasonality, which is in accordance with air temperature and canopy phenology. In addition, all the parameters have significant correlations with the remote sensed vegetation indexes or environment temperature. With parameters estimated with these correlations, the model illustrated fair accuracy both in the validation years and at another alpine grassland ecosystem on Tibetan Plateau. Our results also indicated that the model prediction was less accurate in drought years, implying that soil moisture is an important factor affecting the model performance. Incorporating soil water content into the model would be a critical step for the improvement of the model.

## Introduction

Net ecosystem carbon exchange (NEE) is the balance of gross primary production (GPP) and ecosystem respiration (*R*_eco_). It represents the net exchange of CO_2_ between the atmosphere and terrestrial ecosystems. As a key variable to determine the carbon balance of an ecosystem, NEE has significant importance for studying regional and global carbon cycles. Quantifying NEE between the atmosphere and terrestrial ecosystems is crucial for elucidating the responses of terrestrial ecosystems to global climate change. In recent years, with the development of the eddy covariance technique, it has become possible to assess the ecosystem carbon exchange continuously and steadily for a long term [1]. However, these NEE estimates only represent fluxes at the tower footprint scale, with longitudinal dimensions ranging from a hundred meters to several kilometers relying on homogeneous vegetation and fetch [2]. It thus needs to be scaled up for spatially continuous estimates [3–4].

Satellite can provide consistent and systematic observations of vegetation and ecosystems, and plays an increasing role in estimation of NEE. Many efforts have been made to integrate flux tower data and remote sensing for regional carbon budget research. One approach to estimate NEE with remote sensing products is to develop an empirical regression function between NEE and the remote sensed indices, as well as the climatic variables [5–7]. For this method, the variables used to be related with NEE can be easily accessible at a large spatial scale. It is therefore convenient to be scaled up to the regional scale. However, due to lack of mechanical consideration, the regressive functions linking NEE and the variables are often site-specific, with difficulty to be applied at regional scales [7].

The other approach to estimate NEE is estimating its two components, i.e., gross primary productivity (GPP) and ecosystem respiration (*R*eco) separately, with both of which being estimated with semi-empirical models. For estimating of GPP, the light use efficiency approach had been widely used by several models, such as CASA [8], GLO-PEM [9–10], MODIS-GPP algorithm [11–12], VPM [13–14], EC-LUE [15]. However, problems in the LUE-based GPP models, including biased estimate of the maximum light use efficiency, inconsideration of diffusive radiation, inappropriate representation of soil water constraint, etc., are main hindrances of their applications [15–18]. Based on intensive measurements with eddy covariance systems at global flux tower sites, it is found that GPP can be been approximated as a function of PAR by a light–response curve. In addition, curve-fitting of the light–response by using rectangular hyperbolic functions is generally used as an effective method to gap-fill for missing values in NEE [19]. In recent years, for the purpose of regional applications, some studies attempt to estimate GPP with the light–response functions, the parameters in which were estimated with remote sensed data [20–21]. For estimating *R*eco, well-known exponential functions between soil temperature and *R*eco, e.g., Arrhenius and Van’ t Hoff functions, have been proposed. These models are widely used to estimate *R*eco at site scales when measurements with eddy covariance systems fail [22–24]. However, due to the difficulties in estimating the parameters in the model, very limited studies reported the estimate of *R*eco and hence NEE with remote sensed and climatic data (e.g., [20–21]).

In this study, we proposed a model to estimate NEE with satellite data and climate data using the second approach mentioned above, i.e., the semi-empirical approach. In our model, the component GPP was estimated with a light response curve of a rectangular hyperbola. The component *R*eco was estimated with an exponential function of soil temperature. To test the feasibility of applying our model at regional scales, regression analysis was conducted between the seasonal variations in the model parameters derived from flux tower observations and remote sensed vegetation indexes and climatic factors. Further, the model performance was tested using the parameters estimated with the relationships between the parameters and the vegetation indexes and climate variables.

## Materials and Methods

### Model description

A rectangular hyperbola function is used to depict the relationship between GPP and photosynthetically active radiation (PAR) at the canopy level:

$$\text{GPP}=\frac{\alpha \cdot \beta \cdot \text{PAR}}{\beta +\alpha \cdot \text{PAR}}$$ (1)

where GPP is gross primary productivity (*μmol CO*_*2*_ *m* ^*-2*^ *s* ^*-1*^) *α* is the apparent quantum use efficiency (μmolCO_2_ μmol^-1^PAR). β is the apparent maximum photosynthetic rate (μmol CO_2_ m^-2^ s^-1^). With the method of Goudriaan et al. [25] and Hirose et al. [26], *α* and *β* are further estimated as

$$\alpha =\frac{\left(C_{a}-\tau \right)}{\left(C_{a}+2\tau \right)}\cdot \alpha_{0}$$ (2)

$$\beta =\left(C_{a}-\tau \right)\cdot g_{x}$$ (3)

where *α*_*0*_ (μmol CO_2_ μmol^-1^PAR) is the apparent maximum quantum use efficiency, which is independent from changes in temperature and CO_2_ concentration. As a parameter for the plant growth, *α*_*0*_ should be stable for a specific stand [27]. *g*_*χ*_ is the carboxylation conductance (μmol CO_2_ m^-2^ s^-1^) and can be used to describe the maximum assimilation under low CO_2_ levels [26]. *C*_*a*_ is the ambient CO_2_ concentration of the atmosphere (*μmol mol* ^-1^) and we used 350 *μmol mol* ^-1^ in this study [27]. *τ* is apparent CO_2_ concentration in the chloroplast at the compensation point (*μmol mol* ^-1^), which is estimated as a function of air temperature [28]:

$$\tau \;=\;42.7+1.68\left(T-298\right)+0.0012{\left(T-298\right)}^{2}$$ (4)

Ecosystem respiration (*R*_eco_) is the other component of NEE. In this paper, Van’t Hoff exponential equation was used to describe *R*_eco_ with soil temperature:

$${\text{R}}_{\text{eco}}\text{=}b_{0}\cdot \;e^{b{\text{T}}_{\text{soil}}}$$ (5)

where *b*_0_ is ecosystem respiration rate at reference temperature (here is 0°C), and *b* is a parameter describing the sensitivity of respiration rate to temperature variance.

Based on eqs (1)–(5), NEE, the balance of GPP and *R*_eco_, can be estimated as

$$\text{NEE = }\frac{(C_{a}-\tau )\cdot \alpha_{0}\cdot g_{x}\cdot \text{PAR}}{(C_{a}+2\tau )g_{x}+\text{PAR}\cdot \alpha_{0}}-b_{0}\cdot e^{bT_{soil}}$$ (6)

The parameters in Eq (6) were estimated with the least-squares method. To make it temporally consistent with the 8-day composite MODIS satellite data, we estimated the parameters every 8 days. All the 30-min NEE data within each 8-day were used to invert the parameters. In addition, we also tried to separate Eq (6) into the GPP part and the *R*_eco_ part. Hereafter, with the GPP and *R*_eco_ estimated from eddy covariance system, we inverted the two parameters of photosynthesis (*α*_*0*_ and *g*_*χ*_), and the two parameters of respiration (*b* and *b*_0_), separately. Our results indicated that the second method yielded nearly identical estimation of the four parameters as the method of eq (6). Considering only NEE was measured directly but GPP and Reco were estimated from NEE, in order to minimize the uncertainties from the ‘measurements’, we finally used NEE data only (i.e., Eq (6)) to invert the four parameters. Considering day-to-day variability may exist for the parameters even within 8 days, we thus quantified this variability as the standard error of the optimized value of each 8-day parameterization.

### Sites information

In this study, two alpine grassland ecosystems were selected, with one for model calibration and the other for validation. The first study site is located at 30°29′52′′ N, 91°03′59′′ E with an elevation of 4333 m (a.s.l). The climate is categorized as plateau monsoon climate with characteristics of strong radiation, low air temperature and large diurnal fluctuation. Mean annual air temperature is 1.3°C, with a minimum mean of −10.4°C in January and maximum mean of 10.7°C in July. The mean annual precipitation is 476.8 mm, with 85.1% of which is concentrated from June through August. The vegetation is classified as alpine steppe-meadow. The plant community is dominated by *Stipa capillacea*, *Carex montis-everestii* and *Kobresia pygmaea* with coverage of 10–60% through growing seasons. The soil is classified as meadow soil with sandy loam, with high gravel content of 30% [29].

The other alpine grassland for model validation is located at the Haibei alpine grassland station on the Qinghai-Tibet Plateau (37°40′N, 101°20′E; 3293 m a.s.l.). The mean air temperature is—1.7°C and mean annual precipitation is 580 mm. The dominant species are *Potentilla fruticosa* (Bush cinquefoil), *Kobresia capillifolia*, *Kobresia humilis*, and *Saussurea superba*. During the peak growing seasons, the vegetation reaches a height of about 60 cm, and maximum LAI is about 3 m^2^ m^-2^. The soil is silty clay loam with a heavy clay layer of 0.1–1.0 m in depth [30].

Measurements in this study were taken by the permission of Damxung and Haibei experimental stations.

### Measurements of carbon fluxes and meteorological variables

Open path eddy covariance systems and micrometeorological systems were established at the height of 1.5 m to monitor CO_2_/H_2_O fluxes and environmental conditions at half-hourly intervals. Details of the configuration of the observation systems are available in Hu et al. [30]. When processed the EC flux data, we used a three-angle coordinate rotation approach to align the coordinates with the mean wind [31], and WPL method to adjust density changes resulting from fluctuations in heat and water vapor [32]. Finally, several strategies were adopted to fill missing and rejected data [19].

The measurements of NEE during 2003–2007 at the Damxung site were used to calibrate the model. The measurements during 2008–2009 at the Damxung site and 2004 at the Haibei site were used to test the model performance.

### Satellite data

MODIS land surface reflectance data sets (MOD09A1, 500 m, 8-day) were obtained from the Oak Ridge Distributed Active Archive Center web site (http://www.modis.ornl.gov/modis/index.cfm). Reflectance values of these four spectral bands (blue, red, near infrared (841–875 nm), shortwave infrared (1628-1652nm)) from 2003 to 2009 were used to calculate vegetation indices (NDVI, EVI, and LSWI):

$$\text{NDVI}=\frac{\rho_{nir}-\rho_{red}}{\rho_{nir}+\rho_{red}}$$ (7)

$$\text{EVI}=G\times \frac{\rho_{nir}-\rho_{red}}{L+\rho_{nir}+C_{1}\rho_{red}-C_{2}\rho_{blue}}$$ (8)

$$LSWI=\frac{\rho_{nir}-\rho_{swir}}{\rho_{nir}+\rho_{swir}}$$ (9)

where ρ_nir_, ρ_red_, ρ_blue_, ρ_swir_ is reflectance of near infrared, red, blue, and short infrared bands, respectively. G = 2.5, C1 = 6, C2 = 7.5, and L = 1.

NDVI, EVI and LSWI calculated from MOD09A1 data set were corrected according the QC field in the data set. Then the missing values was filled with the method of Xiao et al[33].

## Results and Discussion

### Seasonal dynamics of NEE and relevant variables

**Fig 1:**
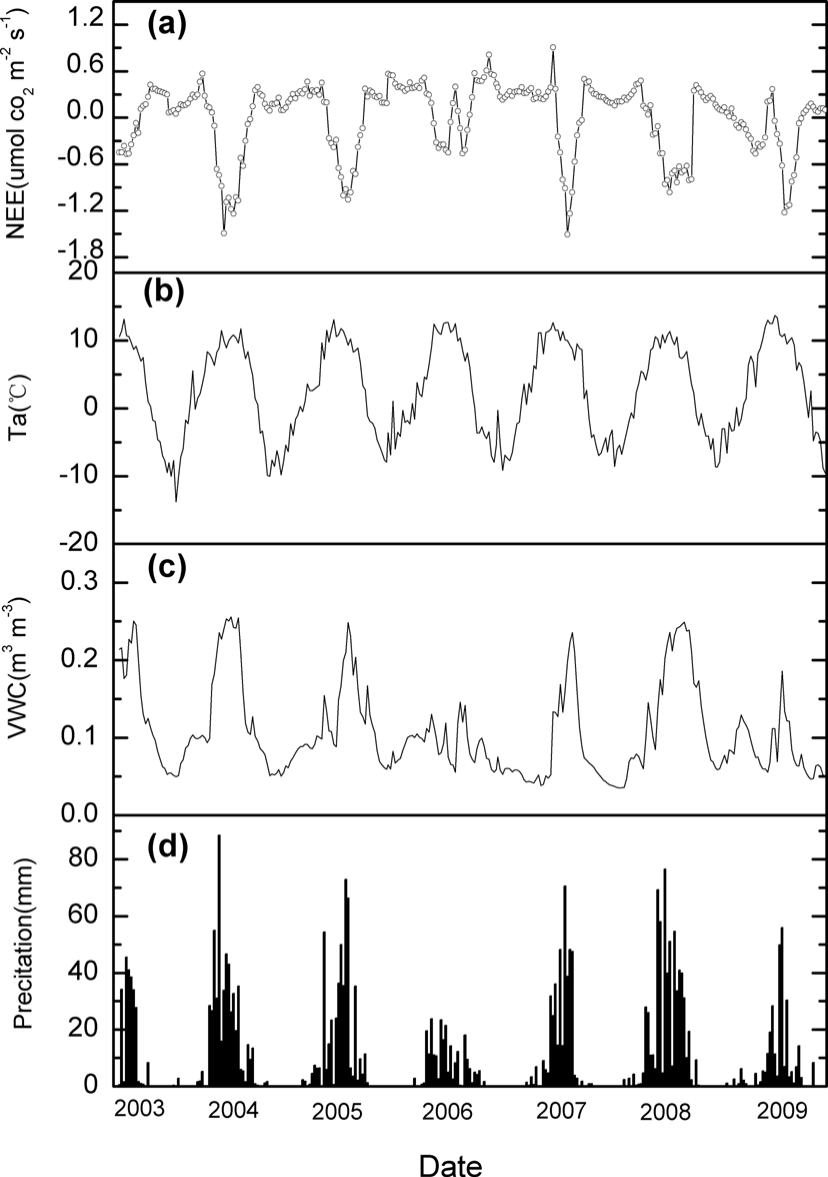
Seasonal dynamics of net ecosystem carbon exchange (NEE), air temperature (*T*_a_), soil volumetric water content (VWC), and precipitation in 2003–2009 at the Damxung site.

Seasonal variations in NEE and relevant environmental variables were presented in Fig 1. NEE and key environmental variables showed apparent seasonal patterns (negative NEE represents carbon uptake and positive NEE represents carbon source). In general, ecosystem carbon uptake increased steadily with the progress of the growing seasons, and decreased after the peaking growing season (the middle of August). Ecosystem carbon uptake (i.e., negative NEE) reached its maximum in the peak growing seasons when the ecosystem experiencing high air temperature and soil water content (Fig 1). Note that the precipitation in 2006 (241 mm) and 2009 (291 mm) was much lower than that in the other years (419-648mm), resulting in lower and more variable soil water content in these two years. Consequently, the NEE in 2006 and 2009 illustrated two or more peaks during the growing seasons. In addition, the total carbon uptake in the two drought years was much lower than that the other years.

**Fig 2:**
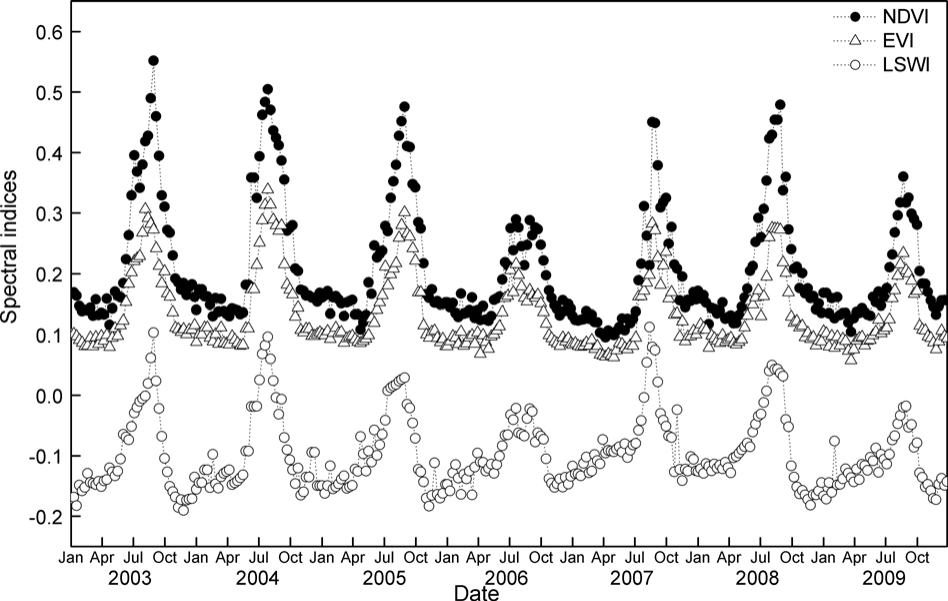
Seasonal dynamics of NDVI, EVI and LSWI in 2003–2009 at the Damxung site.

EVI, NDVI and LSWI at the alpine grassland ecosystem all exhibited similar seasonal patterns, with steady increases with the progress of the growing season and decline in the late growing season (Fig 2). The NDVI and EVI, LSWI time series illustrated similar seasonal cycles, which followed closely to changes in canopy phenology. In addition, the vegetation indices captured the beginning and ending of the growing season well. However, the inter-annual variations in the vegetation index differed in terms of both magnitude and phase. For example, the maximum NDVI value ranges from 0.3 to 0.6, much higher than the maximum EVI values (0.1–0.3). Due to lower precipitation, the indices in 2006 and 2009 were obviously lower than in the other years.

### Seasonal dynamics of the parameters

**Fig 3:**
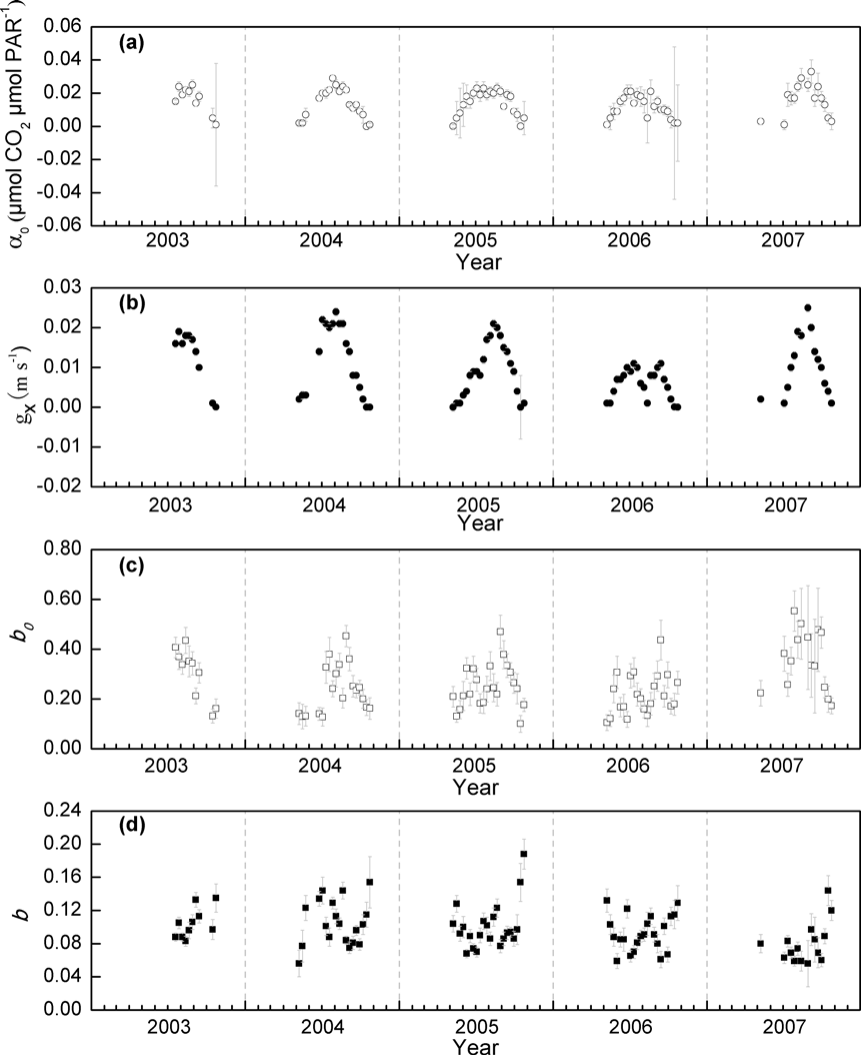
Seasonal variations in the inversed parameters from 2003–2007 at the Damxung site. Error bars represent standard errors.

The seasonal variations in the inverted α_0_, g_x_, *b*_0_ and *b* from July 2003 to October 2007 at the Damxung site were shown in Fig 3. Both *α*_0_, and *g*_x_, the two parameters related to photosynthesis, exhibited similar seasonal patterns. The two parameters increased with the greening-up at the beginning of the growing season, peaked at the middle of the growing season. After the peak growing season, both *α*_0_, and *g*_x_ declined steadily until the end of the growing season. The peak values in 2003–2007 were 0.021–0.033 μmol CO_2_ μmol PAR^-1^ for *α*_0_, and 0.011–0.025 m s^-1^ for *g*_*x*_. The day-to-day variations in the two parameters within each 8-day (i.e., the standard errors) were small in the middle of the growing season, but larger at the beginning and end of the growing seasons.

*b*_0_ showed similar seasonal dynamics with *α*_0_, and *g*_x_, with low values at the beginning and end of the growing seasons, and high values in the peak growing seasons (Fig 3c). However, the fluctuations of *b*_0_ were greater than *α*_0_, and *g*_x_. Meanwhile, *b* showed an opposite seasonal pattern with *b*_0_, with low values in the middle of the growing seasons, and high in the beginning and the end of the growing seasons in a range of 0.056–0.188 (Fig 3d). The opposite seasonal patterns of *b* and *b*_0_ may partly due to the autocorrelation between the two parameters. As Eq (5) illustrates, given a fixed respiration, the two parameters should be negatively correlated. We tried to fix one of the two parameters and invert the other. The results also illustrated that *b* was correlated with soil temperature (when *b*_0_ was fixed), and *b*_0_ was correlated with the vegetation indexes (when *b* was fixed). Considering the model performance will be poorer if we fix either *b* or *b*_0_, we still employee the original method without fixing one of the two parameters.

Our results illustrate high seasonal variations of the parameters influencing the photosynthesis and respiration processes. Similarly, the seasonal variations of maximum photosynthetic rate and quantum use efficiency at canopy scale have also been observed at some temperate forest sites, whereas not clearly observed in subtropical forests [21, 34–35]. These studies suggest that changes in air temperature and phenology mainly control the seasonal changes of canopy maximum photosynthetic rate and quantum use efficiency. In addition, Zhou et al. [36] also found large seasonal variations in maximum carboxylation rate, *V*_cmax_, in several temperate forest sites, which were in accordance with the change of air temperature and LAI. The high seasonality of the parameter highlights the importance of taking into consideration of the variations for the modelling community, whereas fixed values of the parameters are given in the models in most cases.

### Relationships between inversed parameters and spectral indices

**Fig 4:**
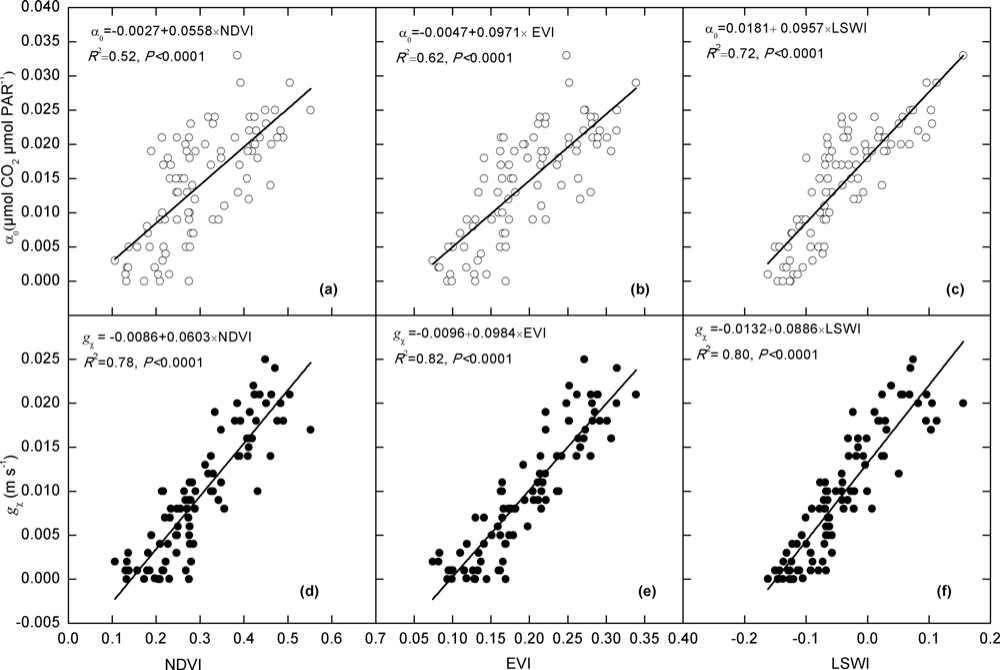
Relationships between inversed *α*_0_ (a-c), *g*_x_ (d-f) and the spectral indices, i.e., NDVI, EVI and LSWI.

All of the three spectral indices illustrated significant linear correlations with *α*_*0*_ and *g*_x_, the two parameters of canopy photosynthesis (P<0.001, Fig 4). These spectral indices could explain more than 51% of the variations in *α*_0_ and 77% in *g*_x_. Among the three spectral indices, LSWI illustrated the highest capacity to explain the variation of *α*_0_ (72.26%). EVI was the most effective index to explain the variations in *g*_x_ (82.09%).

**Fig 5:**
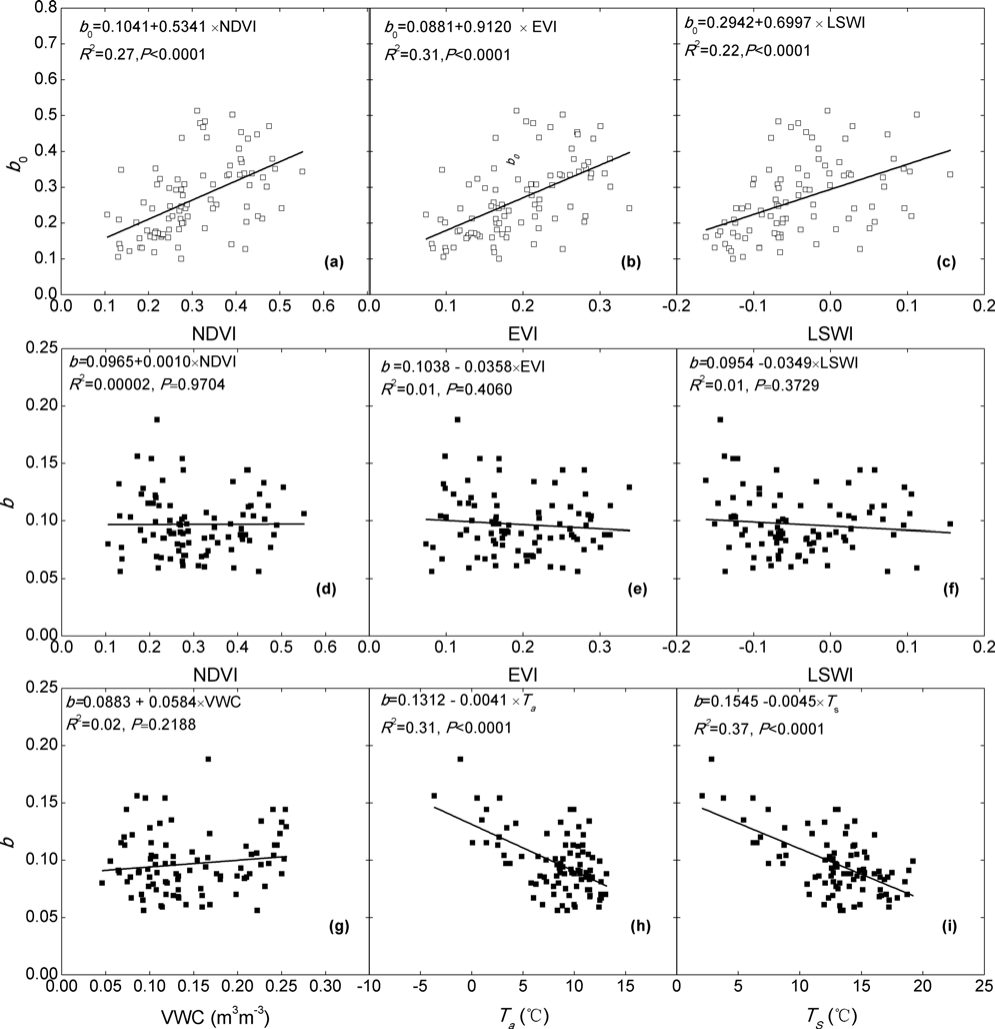
Relationships between inversed *b*, *b*_0_ and the spectral indices (a-f), inversed *b* and environmental variables (g-i).

Similarly, one of the respiration parameters *b*_0_ also exhibited significant correlation with the spectral indices (P<0.001, Fig 5). However, the explanatory capacities of the spectral indices on *b*_0_ (22%-31%) were lower than *α*_0_ and *g*_x_. In comparison, EVI was the best among the three, which could explain 31.4% of the variations in *b*_0_. In contrast, there was no statistically significant correlation between parameter *b* and spectral indices (p>0.05). Instead, *b* was significantly correlated with soil water content, air temperature and soil temperature at the depth of 5 cm (p<0.05), implying that the environmental factors, rather than biotic factors, determines the seasonal variations in this parameter. Soil temperature was the best to explain the variations in *b* with a *R* ^2^ of 0.37.

Our study illustrated significant correlations between the photosynthesis parameters, i.e., *α*_0_ and *g*_x_, and the vegetation indexes. Similarly, Inoue et al. [37] investigated the relationship between seasonal changes in maximum photosynthetic rate, quantum use efficiency and various spectral indices at an irrigated rice field. They reported that the maximum photosynthetic rate was correlated with ratio vegetation index (RVI) and other indices related to the wavelengths in chlorophyll a and b absorption region. In addition, they also found quantum use efficiency was related to the wavelengths in blue region (450 nm) and infrared region (1330 nm). Similarly, Ide et al. [21] found that seasonal variations in both maximum photosynthetic rate and quantum use efficiency were strongly correlated with EVI, independent of the growth stages. Zhou et al. [36] found *V*_cmax_ was significantly correlated with EVI at four temperate forest sites. Possible mechanisms behind the correlations should be related to canopy and leaf properties, e.g., the leaf nitrogen content, which is closely linked with plant photosynthesis capacity. It is reported that leaf pigment content can be estimated from leaf reflectance spectra in visible and near infrared regions [38–39]. Researches have shown that remote sensed reflectance is a useful data source to sense photosynthetic capacity because of the linkage of reflectance with canopy photosynthesis [40–43]. For example, Smith et al. [44] observed a linear relationship between forest productivity and N concentration of canopy leaves independent of vegetation type. Furthermore, canopy N concentration correlates with the absorption rate for chlorophyll in the red region and blue region, and the reflectance rate in the near-infrared region. This indicates the utility of VIs based on these bands, such as EVI, for estimating canopy N content and forest productivity.

In addition to the two photosynthesis parameters, the respiration parameter, *b*_0_ also illustrated significant correlation with the VIs. This result indicates that, besides the environmental factor (e.g., soil temperature), the vegetation itself also modulates ecosystem respiration through affecting the reference respiration. Although there have been some studies find the effect of physiological phenology on plant respiration, this mechanism, however, has not paid enough attention, especially in the modeling community [45]. The negative correlations between *b* and soil temperature implies that the temperature sensitivity of ecosystem respiration decreased with the increasing environmental temperature (Fig 5h and 5i). This is consistent with the reports of decreased temperature sensitivities for soil respiration in other grassland ecosystems under warmer conditions [46–48].

### Performances of the calibrated NEE model

According to the regression analysis of the inverted parameters at the Damxung site, we selected the equations performing the best to estimate each parameter in the validation years and at the validation site (i.e., Haibei). For example, EVI was used to estimate *b*_0_, while soil temperature was used to estimate *b* (Fig 5). The performances of the calibrated NEE model were test with the measurements at Damxung site in 2008–2009, and Haibei site in 2004.

**Fig 6:**
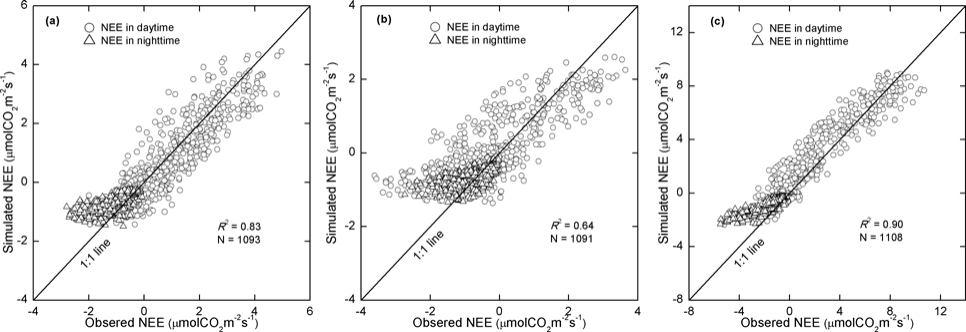
Comparisons of observed NEE via eddy covariance systems with modeled NEE at the Damxung site in 2008 (a), and 2009 (b), and at the Haibei site (c). *R* ^2^ was the coefficient of determination of linear regression between the simulated and observed NEE. N was the total number of the data.

The modeled NEE was in good agreement with the measurements both at the Damxung site in the validation years (i.e., 2008 and 2009) and at the Haibei site (Fig 6). At the Damxung site, the model performed better in 2008 than that in 2009, with a *R* ^2^ of 0.83 and 0.62 in 2008 and 2009, respectively. At the validation site, Haibei, the model also illustrated satisfactory performance, with a *R* ^2^ of 0.90. In comparison, the model performances at nighttime were poorer than that at daytime at both sites and all years (Fig 6). Since the nighttime NEE equals the ecosystem respiration at night, this result implies that a major uncertainty of the model may be from the *R*_eco_ part.

**Fig 7:**
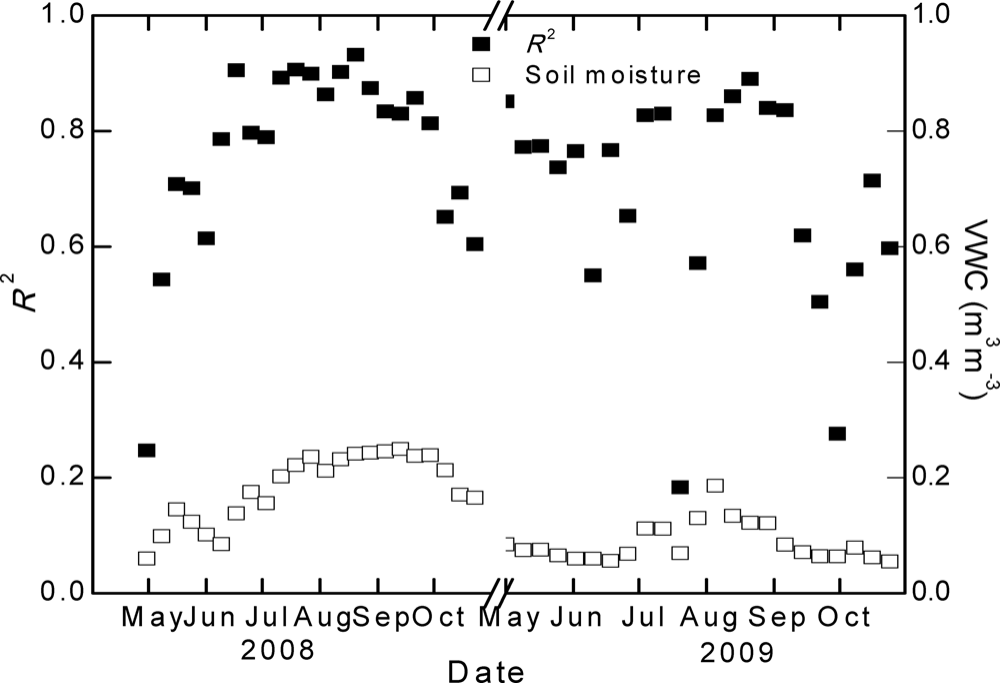
Seasonal variations of the model performance (*R* ^2^) and soil water content during 2008–2009 at the Damxung site.

Apparent seasonal patterns of the model performance were found especially at the Damxung site. The model illustrated high abilities of prediction of NEE in the peak growing seasons and low abilities in the early and late growing seasons (Fig 7). Such seasonality of model performance is in accordance with the variations in soil water content, suggesting that soil moisture is likely an important factor affecting the model performance.

The semi-empirical NEE model developed in this study demonstrated satisfactory predictive capabilities at both sites on Tibetan Plateau. This result confirms the potential of using this scheme to simulate NEE at regional scale. Parameterization is a key issue to apply this model at regional scale. Our study demonstrates that all the parameters could be estimated with either remote sensed VIs or climatic factor, which will facilitate the model’s regional application. Note that only the measurements at one site were used to optimize the model parameters, and only two sites were used for test the model performance in this study. This implies that the current version of the model may not be suitable to estimate the NEE of the whole Tibetan Plateau. To make the model have robust prediction ability at regional scale, measurements at more sites are necessary to develop plant functional type specific regression functions for optimizing the parameters.

Both the seasonality and fluctuation of the model performance are consistent with the variations in soil water content (Fig 7). This implies that the impact of soil moisture might be the main source of model uncertainty. This speculation is supported by the different model performances in the two years at the Damxung site (Fig 6). The model illustrated higher accuracy in the wet year (2008) than the dry year (2009). Soil moisture may affect the estimate of both the GPP component and the *R*_eco_ component. The Michalis-Menten (M-M) equation was used to estimate GPP in this study. M-M is widely used to describe the light-response curve of canopy carbon uptake. However, many studies illustrated that this function could not capture the light response curve with high accuracy when the ecosystem is experiencing drought condition [49]. On the other hand, soil moisture was found as a key regulator of the temperature-sensitivity of soil heterotrophic respiration and an controlling factor of ecosystem respiration at the Damgxun site [29,50]. Owing to the fact that the model performance was poorer at night than in daytime (Fig 6), the *R*_eco_ component, rather than the GPP component, is likely the main source of uncertainty in our model. Using a function with both soil temperature and soil moisture included, would improve the model performance [51].

## Conclusions

Through estimating gross primary productivity with a rectangular hyperbola function of light response, and ecosystem respiration with an exponential function of soil temperature, we proposed a semi-empirical model to estimate NEE with remote sensed vegetation indices and environment variables. Our results indicated that all the inversed parameters of the model illustrated apparent seasonality, which is in accordance with air temperature and canopy phenology. In addition, all the parameters have significant regressive correlations with the remote sensed vegetation indices or environment temperature. With parameters estimated with the regression functions, the model illustrated fair accuracy both in the validation years and at another alpine grassland ecosystem on Tibetan Plateau. The model prediction was less accurate in drought year and at night, implying that soil moisture is an importance factor affecting the model performance. Incorporating soil water content into the model would be critical to improve the model.

## Supporting Information

S1 Dataset Flux and meteorological dataset for Damxung site. (XLSX)

S2 Dataset Flux and meteorological dataset for Haibei site. (XLSX)

S3 Dataset MODIS dataset for Damxung site. (XLSX)

S4 Dataset MODIS dataset for Haibei site. (XLSX)
